# High-pressure activation for the solvent- and catalyst-free syntheses of heterocycles, pharmaceuticals and esters

**DOI:** 10.3762/bjoc.21.102

**Published:** 2025-07-02

**Authors:** Kelsey Plasse, Valerie Wright, Guoshu Xie, R Bernadett Vlocskó, Alexander Lazarev, Béla Török

**Affiliations:** 1 Department of Chemistry, University of Massachusetts Boston, 100 Morrissey Blvd, Boston, MA, USA; 2 Pressure BioSciences Inc., 480 Neponset Street, Unit 10B, Canton, MA, USA

**Keywords:** acetaminophen, acetylsalicylic acid, benzimidazoles, catalyst-free synthesis, cyclization, esters, high hydrostatic pressure, pyrazoles

## Abstract

High hydrostatic pressure (HHP) was found to be an efficient activation method in several catalyst- and solvent-free reactions and has found application for the syntheses of heterocycles and the preparation of active pharmaceutical ingredients (APIs) via acylation and acid- and solvent-free esterification. The reactions were carried out at ambient pressure (control) and under HHP (up to 3.8 kbar) conditions. These representative reactions provided higher yields for the products and HHP enabled truly green processes that are catalyst- and solvent-free, to occur with high yields and producing only non-toxic by-products. A computational study accompanies the experimental data to interpret the outcome of the reactions.

## Introduction

Non-traditional activation methods are one of the major driving forces in green synthesis [1–2]. High hydrostatic pressure (HHP) activation, one of such methods, is based on mechanical compression force. The typical pressure range is 2–20 kbar that is orders of magnitude greater than the conditions traditionally employed in chemistry with pressurized gases (0.01–0.1 kbar). The first reports of using HHP were related to food industry applications [3–4], and later in chemical synthesis [5–6]. While the technique had been known since the late 1800s, and it had become popular in materials science and inorganic synthesis, it has largely gone unnoticed as an activation method for organic synthesis. A large number of applications have been reported in solid state, homogeneous and heterogeneous systems to prepare inorganic compounds and materials [7–9]. In contrast, high pressure organic chemistry, or HHP-initiated organic synthesis is still in its infancy. Despite the recent advances [10–11], the HHP applications in this field are still being developed. The first HHP-assisted organic syntheses were reported in the 1970s [12–13]. Although the mechanism of how pressure enables reactions is not fully elucidated, there is a broad agreement that a decrease in the activation volume (Δ*V*^‡^) and reaction volume (Δ*V*) is the driving force for these reactions [14–16]. A recent computational analysis of high pressure reactions by the extreme pressure polarizable continuum model (XP-PCM) improved the theoretical understanding of the phenomena [17]. In addition, due to the availability of commercially accessible instruments, the applications of HHP in synthetic chemistry have expanded in the past decades. The studied reactions include hydrogenation [18], the addition of enamines to Michael acceptors [19], enantioselective Mannich reactions [20], lipase-catalyzed esterification [21], nitro-aldol [22], Michael [23], and aza-Michael reactions [24–25], Diels–Alder reactions [26–27] and Friedel–Crafts alkylation of indoles [28]. Many high pressure reactions were applied in natural product synthesis [29]. The high pressure protocol was even applied in the elegant syntheses of platencin [30], and steroid derivatives [31]. Despite these encouraging applications, there is a broad area to be developed for the advancement of green synthesis efforts.

**Figure 1:**
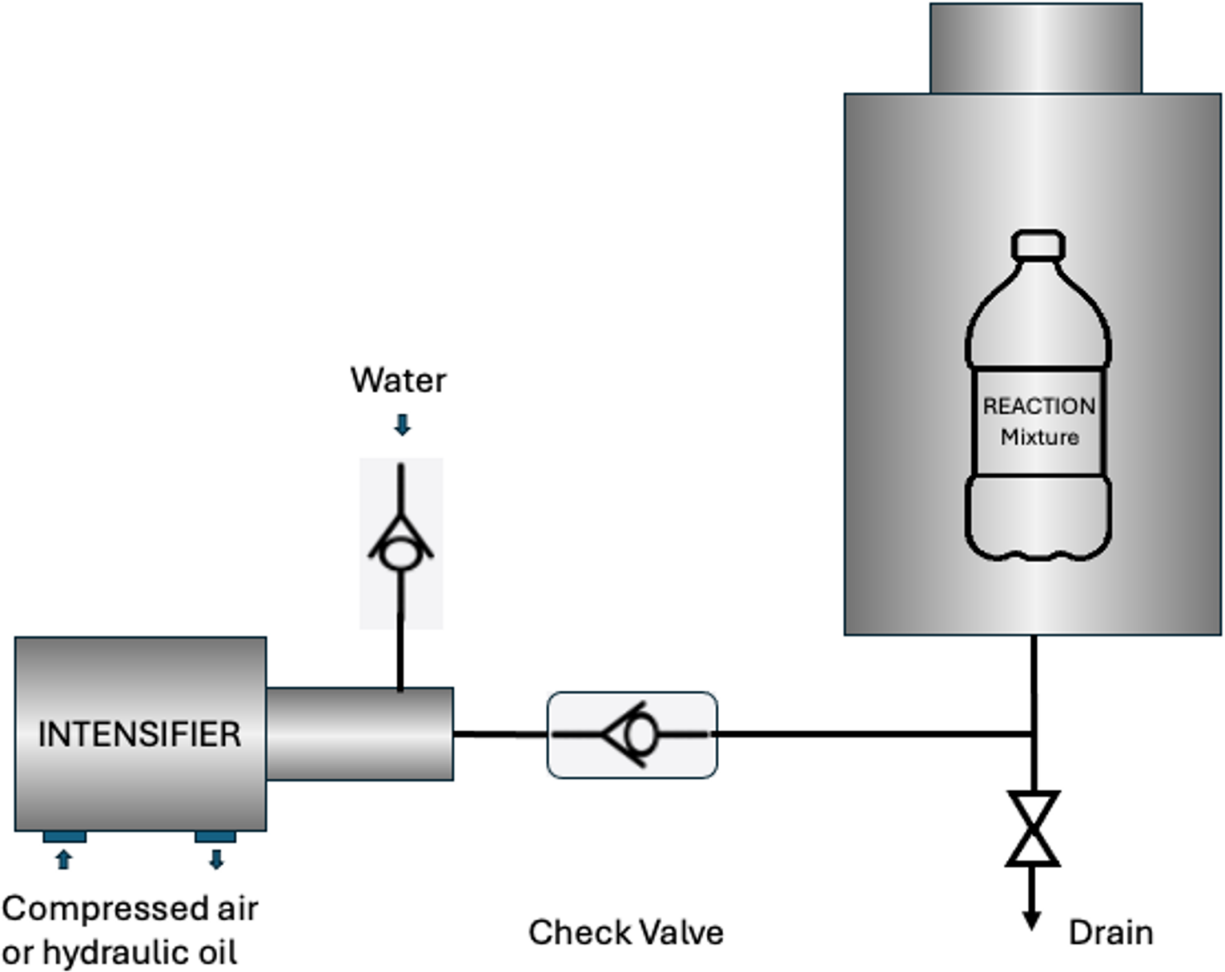
Simplified schematic rendering of a high hydrostatic pressure reactor.

Continuing our program on developing environmentally benign synthetic methods [32–35], especially in the realm of high pressure chemistry [36–37], in the present work we demonstrate a variety of new, catalyst- and additional solvent-free applications of HHP to develop green synthesis methods. Here, we describe several cyclization reactions for the preparation of a variety of important heterocycles, the synthesis of well-known APIs, such as acetaminophen and acetylsalicylic acid, a variety of esterification reactions and the successful scale up (up to 100 g scale) of the Paal–Knorr reaction. The use of HHP appears to provide several advantages, for example, resulting in higher reaction rates. Given that every reaction in the present work has been carried out without a catalyst, in many instances this contrast is even greater since the non-pressurized reactions simply did not take place. In other applications when ambient pressure reactions yielded product, HHP still offered a reasonable increase in rates and thus, in yields. In addition to the synthetic advantages, the benefits HHP offers can also translate to green chemistry. The use of HHP extends the scope of solvent-, reagent- and catalyst-free reactions that significantly reduce workup and waste generation. Using water as pressure transmitting fluid, the reaction vessel is immersed in water minimizing fire hazard during the reactions. Finally, most procedures can be carried out at ambient temperature, improving safety and energy efficiency. Energy efficiency is also supported by the nature of the reactions; although pressurizing the system requires energy, once the system is pressurized it does not need energy to maintain it. This can result in remarkable energy saving especially in long reactions. In order to aid the understanding of this technique a schematic design of a high hydrostatic pressure instrument is depicted in Figure 1.

The core of the instrument is the intensifier which generates the required pressure by amplifying the relatively low pressure (about 140 psi) supplied by an air compressor. The intensifier pressurizes the water in the pressure chamber where the sealed samples are placed. Water is used as a pressure transmitting medium that has relatively low compressibility, readily available, and non-toxic.

## Results and Discussion

### HHP-assisted synthesis of 1,3-dihydrobenzimidazoles

Heterocycles are extremely versatile compounds and are used as building blocks for fine chemicals and conducting polymers or scaffolds for drug synthesis. Among them, 1,3-dihydrobenzimidazoles are widely found in many materials, drug candidates, and catalysts. For instance, they can be used in organic light-emitting diodes (OLEDs) [38], as water-soluble antitrypanosomatid agents [39], or in the synthesis of imidazole-based N-heterocyclic carbene (NHC)–CuCl complexes [40]. However, their synthesis is often tainted by the use of toxic reagents and solvents. In addition, when *o*-phenylenediamine reacts with ketones, the common catalytic methods yield benzodiazepine products [41]. In our case the reaction of *o*-phenylenediamine and acetone was selected as a model reaction. The optimization of the reaction conditions is summarized in Table 1. All reactions were carried out at room temperature without involving any catalyst or additional solvent. While *o*-phenylenediamine is solid, it dissolves well in the reactant acetone, thus the actual reaction mixture remains in a liquid state, making it ideal for HHP-assisted reactions. The first reaction in the optimization effort was the control experiment at atmospheric pressure. Under these conditions, no product formation was observed even after 10 h reaction time. However, 8% of products were generated when 2.8 kbar was applied after only 1 h reaction time. By gradually increasing both pressure and reaction time, higher yields were obtained. The data shows that increasing the pressure up to 3.8 kbar nearly linearly increases the product yield. Meanwhile, the pressure effect appears to work synergistically with reaction time.

**Table 1:** Optimization of the HHP-initiated synthesis of 1,3-dihydro-2,2-dimethylbenzimidazole (**3a**).

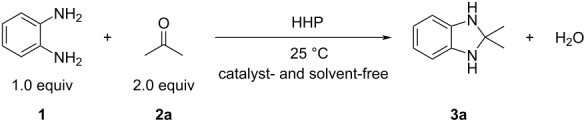			

Entry	Pressure (kbar)	Time (h)	Yield (%)^a^

1	0.001	10	0
2	2.8	1	8
3	3.4	1	11
4	3.8	1	25
5	3.8	2	32
6	3.8	10	90
7^b^	3.8	10	54

^a^GC yields; ^b^1.0 equiv acetone.

**Scheme 1:**
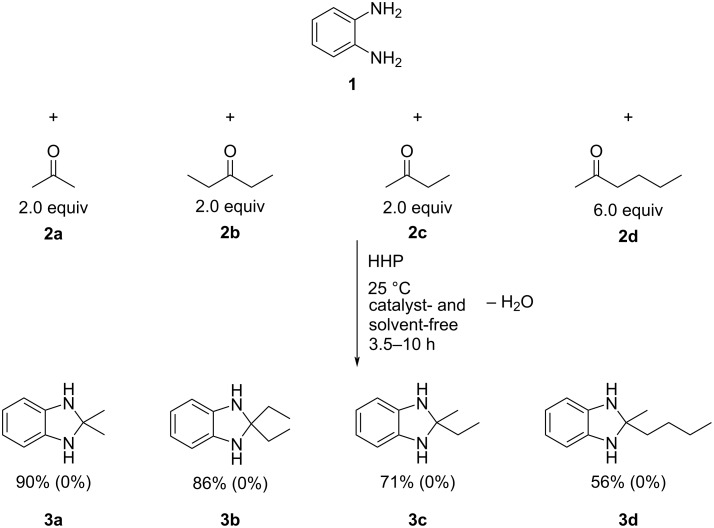
High pressure-initiated synthesis of 1,3-dihydrobenzimidazoles **3a**–**d**. The yields are GC yields and the numbers in parentheses show the yields of the control reactions (1 bar pressure).

The optimization data indicate that the use of HHP resulted in the formation of 1,3-dihydro-2,2-dimethylbenzimidazole (**3a**) in excellent yield (90%) whereas, in contrast, the ambient pressure reaction did not provide any product. The reaction appears to be applicable for other substrates as well, although the yields were lower, which is mainly due to the decomposition of the products (Scheme 1).

### HHP-assisted cyclization of chalcones with hydrazines for the synthesis of pyrazoles

**Figure 2:**
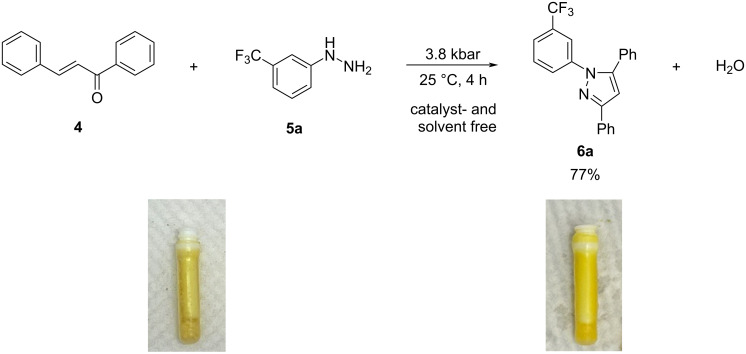
Illustration of the cyclization reaction between chalcone (**4**) and 3-(trifluoromethyl)phenylhydrazine (**5a**) under 3.8 kbar pressure at small (≈150–200 mg) scale reactions.

The cyclization of 1,3-bifunctional compounds, such chalcones, with two N-containing substrates can yield a variety of valuable heterocycles, including pyrazoles. Accordingly, their syntheses attracted extensive interest, and several green procedures have been published [42]. Many of these reactions still apply catalysts and organic solvents, thus the development of catalyst- and solvent-free processes is desirable. Chalcones are a privileged scaffold in medicinal chemistry and are used for the synthesis of a multitude of products [43]. The cyclization between chalcones and hydrazines usually occur via a C=O/NH_2_ condensation and a subsequent NH addition to the C–C double bond, that most commonly require some form of catalysis. Thus, developing catalyst-free processes presents significant challenges, although there are few successful examples in the literature [44]. Similar to the dihydrobenzimidazoles above, the investigations here also started with an optimization of the reaction conditions, including substrate ratio, pressure, and reaction time. The cyclization of chalcone with 3-(trifluoromethyl)phenylhydrazine was selected as a test reaction. This reaction is relatively easy to follow even visually. Once the reaction mixture is subjected to 3.8 kbar pressure, the originally liquid mixture turns to a semisolid, viscous oily product, as shown in Figure 2. The numerical values are presented in Table 2.

**Table 2:** Optimization of the reaction conditions for the synthesis of 3,5-diphenyl-1-(3-(trifluoromethyl)phenyl)-1*H*-pyrazole (**6a**) under ambient pressure (0.001 kbar) and high hydrostatic pressure from chalcone (**4**) and 3-(trifluoromethyl)phenylhydrazine (**5a**).

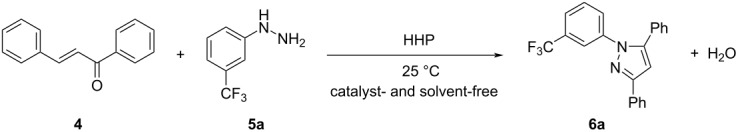				

Entry	Molar ratio (chalcone/hydrazine)	Pressure (kbar)	Time (h)	Yield (%)^a^

1	1:2	0.001	4	12
2	1:1	0.7	1	14
3	1:2	1.4	1	12
4	1:1	2.1	1	12
5	1:1	2.8	1	14
6	1:1	3.8	1	56
7	1:3	3.8	1	57
8	1:1	3.8	4	60
9	1:2	3.8	4	78

^a^GC yield.

**Scheme 2:**
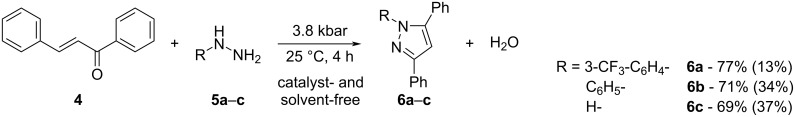
High pressure-initiated catalyst- and solvent-free synthesis of pyrazoles **6a**–**c** from chalcone (**4**) and hydrazines **5a**–**c**. The GC yields at high pressure are shown for each compound, and the numbers in parentheses represent the yields of the respective control reactions at 1 bar pressure. In the case of the unsubstituted hydrazine (NH_2_–NH_2_), the non-aromatic product, dihydropyrazole, formed.

The data shows that using excess hydrazine under pressure of 3.8 kbar resulted in the best performance. Based on the optimized data several compounds were subjected to those conditions to provide a representative scope of the reactions and the results are summarized in Scheme 2. For comparison, the yields obtained under ambient pressure (1 bar) are provided in parentheses.

The data show that the reactions readily occur at room temperature providing good yields. Although the reactions do occur at ambient pressure, the pressurized reactions generally provide higher yields under the otherwise same conditions. It also appears that the reactivity of the hydrazine plays a significant role. In the case of the highly reactive hydrazine, the positive effect of HHP is somewhat diminished and only about 10% increase in yield was observed. In contrast, when using substituted phenylhydrazines of lower reactivity HHP results in more significant benefits, for example a nearly 70% higher yield in the case of the chalcone/3-(trifluoromethyl)phenylhydrazine reaction (Scheme 2).

### HHP-assisted acylation of NH and OH groups: synthesis of acetaminophen (paracetamol) and acetylsalicylic acid

Tylenol^®^ and Aspirin^®^ are two popular drugs used as pain killers as well as antipyretics [45]. Although their industrial production is straightforward, these syntheses include the use of catalysts and solvents that must be neutralized and recycled. Given the large scale these compounds are prepared at, even a small green improvement in their synthesis might yield great environmental, as well as financial benefits. Thus, these two compounds were selected as model compounds to investigate the potential benefits of the HHP-assisted synthesis. Similar to the other reactions, preliminary optimization experiments have been carried out as described in Table 3.

**Table 3:** Optimization of the high pressure-assisted catalyst- and additional solvent-free synthesis of acetylsalicylic acid (**9**).

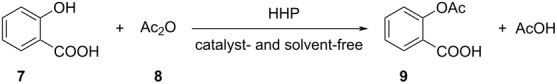					

Entry	Molar ratio (**7**/**8**)	Pressure (kbar)	Time (h)	Temperature (°C)	Yield (%)^a^

1	1:2	0.001	0.5	80	30
2	1:1	3.8	1.5	25	6
3	1:2	3.8	1.5	25	8
4	1:1	3.8	12	25	24
5	1:2	3.8	12	25	36
6	1:1	3.8	2	50	36
7	1:2	3.8	2	50	41
8	1:2	3.8	2	80	64
9	1:2	2.8	0.5^b^	80	100

^a^GC yield; ^b^30 × 1 min cycles, with 5 s decompression.

**Figure 3:**
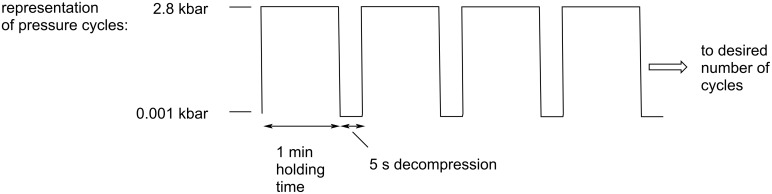
Schematic representation of the cycling experiments: the major variables are the applied pressure, the holding time, the length of decompression and the number of cycles in the sequence.

The results clearly indicate that the product yields improve under high pressure conditions. The best performance was obtained under pressure cycling conditions as illustrated in Figure 3.

It has been observed that pressure cycling resulted in a quantitative yield after 30 1-min cycles (Table 3, entry 9), that would account for 30 min reaction under pressure, and provides a significantly better yields than the reaction conducted under ambient pressure (30%, Table 3, entry 1) or a 2 h reaction at static higher pressure (64%, Table 3, entry 8). While the exact nature of this phenomenon is not fully understood, it is hypothesized that the pressure cycling protocol causes periodic change in the volume of the reaction vessel that could lead to molecular re-alignments during compression and decompression steps that are beneficial for reaction kinetics.

**Scheme 3:**
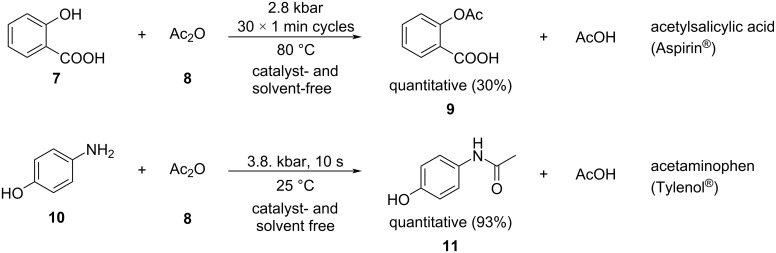
High pressure-initiated synthesis of the active pharmaceutical ingredients in Tylenol® and Aspirin®. The yields refer to GC yields and the numbers in parentheses represent the yields of the control reactions at 1 bar pressure.

Under optimized conditions, the products were isolated with good to excellent yields under a catalyst- and solvent-free environment (Scheme 3). The data in Scheme 3 illustrate that HHP provides some advantage in both reactions, more evident in the case of OH acylation. The NH_2_ acylation occurred much more rapidly in 10 s at room temperature, while also generating the product nearly quantitatively. The OH group is significantly less reactive under such conditions; therefore, the use of pressure resulted in a more substantial improvement in the OH acylation reaction.

### HHP-assisted esterification of alcohols: synthesis of fragrances

Esterification is one of the most common organic reactions and there are a multitude of processes available. However, most require some form of catalysis from simple acids to metal catalysts [46]. Similar to the previous examples, the first step was the optimization of the conditions using the esterification of benzyl alcohol (**12a**) with acetic anhydride (**8**) and acetic acid (**13**), respectively. The optimization data are summarized in Table 4.

**Table 4:** Optimization of the HHP-assisted catalyst-free esterification of benzyl alcohol (**12a**).

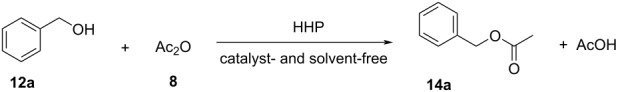					

Entry	Molar ratio (**12a**/**8**)	Pressure (kbar)	Time (h)	Temperature (°C)	Yield (%)^a^

1	1:2	0.001	1	80	52
2	1:1	3.8	2	50	38
3	1:2	3.8	2	50	46
4	1:1	3.8	2	80	52
5	1:2	3.8	2	80	100
6	1:3	3.8	2	80	100
7	1:1	3.8	1	80	92
8	1:2	3.8	1	80	80
9	1:3	3.8	1	80	100

^a^GC yield.

**Scheme 4:**
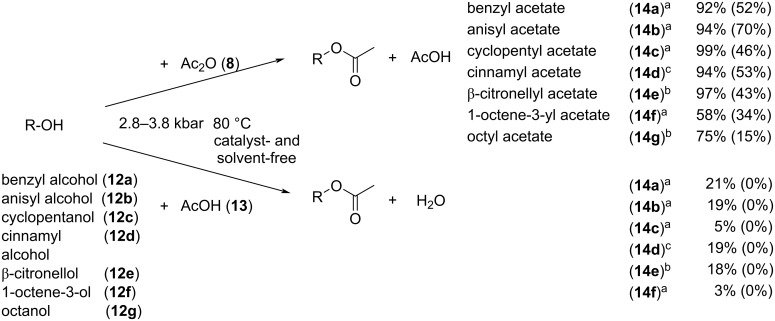
High pressure-initiated esterification of alcohols **12a**–**g** in a catalyst- and additional solvent-free reactions with either acetic anhydride (**8**) or acetic acid (**13**). All reactions were carried out at 80 °C and at (a) 3.8 kbar, 1 h, (b) 2.8 kbar, 30 × 1 min cycles, (c) 2.8 kbar, 40 × 1 min cycles. The yields were determined by GC.

The data indicates that the use of pressure is beneficial for the esterification reaction. The ambient pressure reaction only yields 52% product (Table 4, entry 1) compared to the 92% obtained under 3.8 kbar. Increasing the ROH/Ac_2_O ratio (1:3, Table 4, entry 9) or the reaction time (2 h, Table 4, entry 5) under pressure yielded quantitative product formation. The optimized conditions allowed the extension of the protocol and a selection of alcohols was investigated. The results are illustrated in Scheme 4. For comparison, results obtained from reactions at ambient pressure are given in parentheses.

As shown in Table 4 and Scheme 4, the HHP reactions occurred at 50 °C with low to moderate yields, and with nearly quantitative yields at 80 °C. HHP reactions afforded the products in higher yields than those of the control experiments under the same conditions (time, temp. etc.) but only at 1 bar (atmospheric) pressure. It appears that the pressurized reactions provided about 40–60% yield improvements in the same timeframe as the controls. The reactions gave significantly higher yields when using Ac_2_O, than with AcOH. However, while the reactions with acetic acid after 1 h under pressure all yielded the expected products to some extent, no product formation was observed at the ambient pressure control reactions.

Unlike water and a few elements (silicon, bismuth, gallium, etc.) that tend to form tetrahedral crystal lattices and exhibit lower density upon freezing, most organic compounds are expected to form crystals of higher density, compared to their liquid state. However, phase transitions of the vast majority of organic compounds at high pressure are not yet adequately studied. Data on their behaviour when pressurized are not readily available, except for some long chain aliphatic hydrocarbons, due to their propensity to sedimentation in pressurized oil pipelines. Attempts to correlate known phase transition parameters of reactants and products at atmospheric pressure, such as their respective melting point and boiling point at 1 bar, have not revealed any conclusive trends with respect to the observed reaction yields shown in Scheme 4. While Ac_2_O is generally more reactive than AcOH, it is conceivable that the significant difference in esterification yields with acetic anhydride vs. acetic acid is further enhanced due to acetic acid forming solid crystalline state during pressurization even at 80 °C. However, the reaction rate with AcOH is still much improved under high pressure, as none of the alcohols formed any product under 1 bar pressure.

### Scale up of the HHP-assisted reactions

**Scheme 5:**
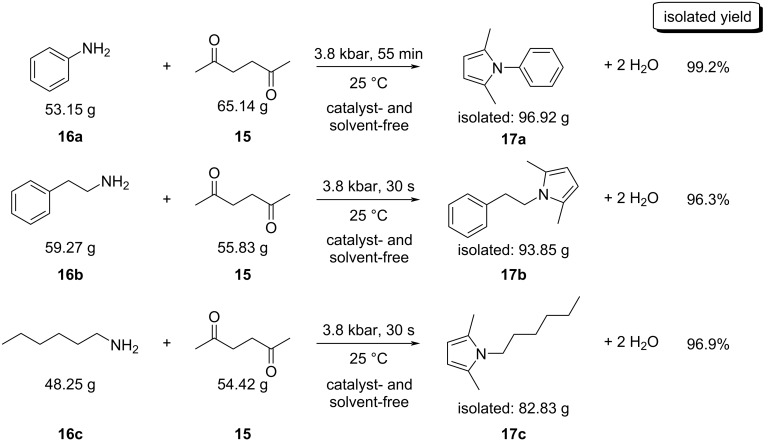
High pressure-initiated large scale syntheses of *N*-aryl- and *N*-alkylpyrroles at about 100 g scale.

The above protocols were carried out at a small scale (mg to g) and thus, an effort was made to investigate the scale-up potential of the HHP-assisted method. Although the HHP-assisted organic syntheses are relatively rare, the large (often ton) scale-capable equipment is readily available in the food industry. Accordingly, industrial level organic syntheses are attainable in a large scale. In an earlier work we already demonstrated that such reactions, e.g. the HHP-assisted Paal–Knorr reaction can be easily scaled up to a multigram level [36]. Although the reaction occurred in catalyst-free systems without pressure the reaction times were 24 h or more [47]. Thus it was attempted to provide some examples for the further scale-up of the Paal–Knorr reaction in a much larger scale, at this time, up to a 100 g level. The experiments were carried out in a 5-L capacity high pressure instrument (Pressure BioSciences) using commercially available low-density polyethylene (LDPE) bottles as reaction vessels and afforded the products with good yields (Scheme 5).

Although the chemically inert nature of reaction vessels is essential, the ductile amorphous property of the material is also of the utmost importance, making the vessel flexible enough to handle the decreasing volume of the reaction mixture under pressure: contrary to a common misconception that liquids are incompressible, a significant (5–12%) decrease in the volume is generally observed at the pressure levels studied here.

The results of these experiments show that the reaction scale can be readily increased to the ≈100 gram level, suggesting that achieving these reactions at even higher scales is reasonable. The reactions occurred in a similar fashion as they did at the mg scale [36] and all reactions produced higher than 96% isolated yield of the product. Where the two alkyl amines were used, the reactions were complete within 30 s of reaction time. Even the less reactive aniline gave nearly quantitative isolated yields in less than an hour, while the 1 bar control reaction yielded no detectable products. In addition to the excellent yields, the products were isolated without any purification, using a simple air-drying process to remove the water that forms as a by-product during the cyclization–aromatization sequence. The gas chromatograms of the three crude products appeared essentially 100% pure, neither starting material nor other by-products were observed.

In order to explain the outcome of the above reactions, the reaction volume data (Δ*V*) was calculated for each reaction. Gaussian 09 was used to carry out the calculations at the b3lyp/6-311++g(d,p) level of theory. The molecular volume of each reactant and product (including all by-products that are part of the reaction scheme) was calculated and the overall volume difference of the starting materials and the products was determined. The obtained values are summarized in Tables 5–10.

**Table 5:** Molecular volumes of the reactants and products and the reaction volume (Δ*V*) for the reaction of *o*-phenylenediamine with ketones to form 1,3-dihydrobenzimidazoles.

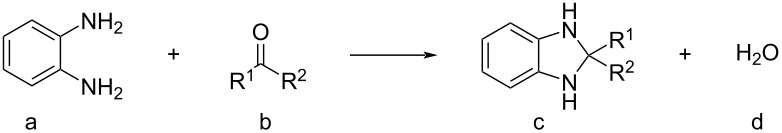							

Entry	R^1^	R^2^	*V*_a_ (cm^3^/mol)	*V*_b_ (cm^3^/mol)	*V*_c_ (cm^3^/mol)	*V*_d_ (cm^3^/mol)	∆*V*_(c + d) − (a + b)_ (cm^3^/mol)

1	Me	Me	94	60	130	18	−6
2	Et	Et	94	81	150	18	−7
3	Me	Et	94	66	140	18	−2
4	Me	*n*-Bu	94	100	140	18	−36

**Table 6:** Molecular volumes of the reactants and products and the reaction volume (Δ*V*) for the reaction of chalcone with hydrazines to form pyrazoles.

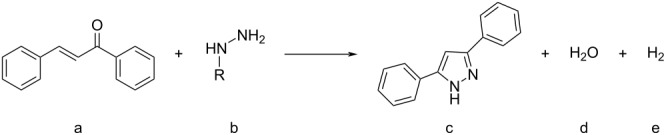							

Entry	R	*V*_a_ (cm^3^/mol)	*V*_b_ (cm^3^/mol)	*V*_c_ (cm^3^/mol)	*V*_d_ (cm^3^/mol)	*V*_e_ (cm^3^/mol)	∆*V*_(c + d) − (a + b)_ (cm^3^/mol)

1	3-CF_3_-C_6_H_4_-	180	110	260	18	13	1
2	C_6_H_5_-	180	92	230	18	13	−11
3	H-	180	37	160	18	13	−26

**Table 7:** Molecular volumes of the reactants and products and the reaction volume (Δ*V*) for the reaction of chalcone with hydrazines to form dihydropyrazoles.

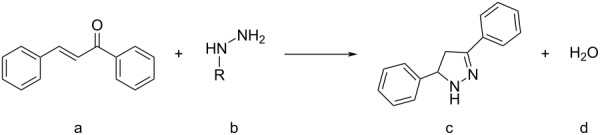						

Entry	R	*V*_a_ (cm^3^/mol)	*V*_b_ (cm^3^/mol)	*V*_c_ (cm^3^/mol)	*V*_d_ (cm^3^/mol)	∆*V*_(c + d)−(a + b)_ (cm^3^/mol)

1	3-CF_3_-C_6_H_4_-	180	110	270	18	−2
2	C_6_H_5_-	180	92	230	18	−24
3	H-	180	37	190	18	−9

**Table 8:** Molecular volumes of the reactants and products and the reaction volume (Δ*V*) for the esterification reaction of alcohols with acetic anhydride.

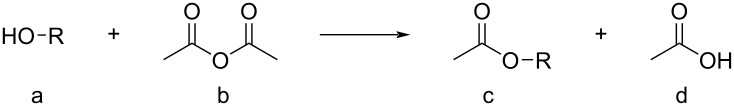							

Entry	alcohol	R	*V*_a_ (cm^3^/mol)	*V*_b_ (cm^3^/mol)	*V*_c_ (cm^3^/mol)	*V*_d_ (cm^3^/mol)	∆*V*_(c + d) − (a + b)_ (cm^3^/mol)

1	benzyl alcohol	-CH_2_C_6_H_5_	81	73	120	38	4
2	anisyl alcohol	-CH_2_C_6_H_4_OCH_3_	130	73	140	38	−25
3	cyclopentanol	-C_5_H_9_	76	73	100	38	−11
4	cinnamyl alcohol	C_6_H_5_CH=CHCH₂-	120	73	140	38	_−_15
5	β-citronellol	C_9_H_19_CH_2_-	160	73	180	38	−15
6	1-octene-3-ol	H_2_C=CHCH_2_CH-(CH_2_)_3_CH_3_	120	73	140	38	−15
7	octanol	CH_3_(CH_2_)_7_-	140	73	190	38	−25

**Table 9:** Molecular volumes of the reactants and products and the reaction volume (Δ*V*) for the esterification reaction of alcohols with acetic acid.

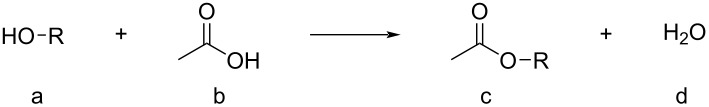							

Entry	alcohol	R	*V*_a_ (cm^3^/mol)	*V*_b_ (cm^3^/mol)	*V*_c_ (cm^3^/mol)	*V*_d_ (cm^3^/mol)	∆*V*_(c + d) − (a + b)_ (cm^3^/mol)

1	benzyl alcohol	-CH_2_C_6_H_5_	81	38	120	18	19
2	anisyl alcohol	-CH_2_C_6_H_4_OCH_3_	130	38	140	18	−10
3	cyclopentanol	-C_5_H_9_	76	38	100	18	4
4	cinnamyl alcohol	C_6_H_5_CH=CHCH₂-	120	38	140	18	0
5	β-citronellol	C_9_H_19_CH_2_-	160	38	180	18	0
6	1-octene-3-ol	H_2_C=CHCH_2_CH-(CH_2_)_3_CH_3_	120	38	140	18	0
7	octanol	CH_3_(CH_2_)_7_-	140	38	190	18	−10

**Table 10:** Molecular volumes of the reactants and products and the reaction volume (Δ*V*) for the Paal–Knorr reaction of amines with 2,5-hexanedione.

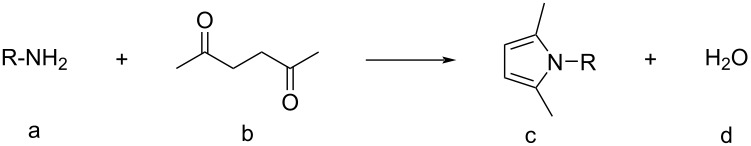						

Entry	R	V_a_ (cm^3^/mol)	V_b_ (cm^3^/mol)	V_c_ (cm^3^/mol)	V_d_ (cm^3^/mol)	∆V_(c + d)-(a + b)_ (cm^3^/mol)

1	C_6_H_5_-(CH_2_)_2_	130	93	160	18	−45
2	C_6_H_5_-	90	93	150	18	−15
3	CH_3_-(CH_2_)_5_-	110	93	170	18	−15

The data in Tables 5–10 indicate that the reaction volumes appear to be in the negative region for the large majority of examples, some being 0 or a small positive number. Based on the theoretical models briefly discussed in the introduction, the negative Δ*V* values suggest that these reactions respond well to pressurized conditions by improved yields, and increased reaction rates. One must note, however, that Δ*V* is just one of the characteristic descriptors of high pressure reactions, and full analysis can be obtained by also evaluating the activation volume (Δ*V*^‡^) data. Considering that all of the above studied reactions are multistep processes with several elementary steps, the determination of Δ*V*^‡^ requires a detailed theoretical reaction mechanism study that is beyond the scope of the current work. Nonetheless, since the Δ*V* values provide clear support for the beneficial effect of pressure, it is reasonable to predict that the activation volumes are also either negative or near zero to explain to observed experimental data.

## Conclusion

The high hydrostatic pressure activation has been successfully applied to several reactions; cyclizations of various types, acylations and esterifications. In addition to being efficient at a small scale, the scale up of the Paal–Knorr reaction was also achieved. Compared to the traditional benchtop alternatives the HHP-assisted approach provided several benefits, such as (i) improved reactions rates and yields in catalyst and solvent-free reactions, which enables easy isolation, and simplified work-up procedure; (ii) the neat reactions of the substrates eliminates solvents and thus reduces environmental and hazard impact; (iii) the HHP conditions enable many reactions to proceed at ambient temperature, resulting in convenient, safe, and energy-efficient protocols; and finally, (iv) HHP instruments allow broadly tuneable procedures that include modifying pressure, temperature, reaction time, or pressure cycles to ensure easy process optimization. Based on our earlier publications and the data presented here, it is reasonable to predict that the high-pressure activation strategy will gain broad application possibilities in the future.

## Experimental

### Materials and methods

Materials: All substrates (aldehydes, hydrazones, alcohols, *p*-aminophenol, salicylic acid, acetic anhydride, acetic acid, hexan-2,5-dione, aniline, hexylamine and 2-phenylethylamine) were purchased from Aldrich and used without any purification. Ethyl acetate, used to dissolve the products for GC–MS analysis (minimum purity of 99.5%) was purchased from ThermoFisher Scientific. The small scale reactor tubes “PCT MicroTubes” were made of FEP and PTFE Teflon^®^ and obtained from Pressure BioSciences Inc., while the larger scale bulbs and bottles used as reaction vessels were made of low-density polyethylene and purchased from ThermoFisher Scientific.

Analysis: All compounds are common, known entities and they were identified based on their high-resolution mass spectra. The mass spectrometric identification and purity determination of the products have been carried out by using an Agilent 7250 GC-QTOF mass spectrometer operated in electron impact ionization (EI, 70 eV) mode using a 30m long DB-5 type column (J&W Scientific).

### General protocols for HHP reactions

The high pressure syntheses can be carried out in two different ways, in manners analogous to ref. [37]. A bench-top Barocycler 2320EXT was used to initiate HHP reactions. (i) *Constant pressure* means that the system is pressurized to the desired pressure that is maintained through the reaction and decompressed, when complete. (ii) *Pressure cycling.* Another, HHP approach is the pressure cycling, when compression–decompression cycles are repeated. In these investigations, 2.8 kbar was selected as applied pressure based on the optimization experiments (Table 3).

#### General procedure for the catalyst and solvent-free reactions of chalcone with hydrazines under HHP conditions

To a 150 µL high-pressure FEP reaction tube was added chalcone (52.0 mg, 0.25 mmol) and 3-(trifluoromethyl)phenylhydrazine (65 mg, 0.37 mmol) which could just fill up the entire reaction tube, and the tube was sealed by a PTFE PCT MicroCap and secured in the MicroTube carrier to ensure the secure seal. Then, the tube was placed in the chamber compartment of a Barocycler 2320EXT (Pressure BioSciences Inc.). The chamber was filled with water and pressurized up to the desired pressure and the pressure was maintained for the predetermined time. After removing the reaction tube, from the pressure chamber, the product was dissolved in ethyl acetate and the yield was determined by an Agilent 7250 GC-QTOF mass spectrometer operated in electron impact ionization (EI, 70 eV) mode using a 30 m long DB-5 type column (J&W Scientific).

#### General procedure for the high hydrostatic pressure-initiated catalyst and solvent-free synthesis of 1,3-dihydrobenzimidazoles

The appropriate amount of the reactants was added into a 3.5 mL volume LDPE reaction vial. The well-sealed reaction mixtures were placed into the chamber. Water is filled into the HHP vessel and pressurized to the desired pressure up to 3.8 kbar. After the reactions are completed, the pressure was released and the reaction products are isolated.

#### General procedure for the catalyst and solvent-free acylation of hydroxyl compounds and *p*-aminophenol under HHP conditions

To a 150 µL high-pressure FEP reaction tube was added the corresponding hydroxy compound or *p*-aminophenol (0.25 mmol) and acetic anhydride or acetic acid in a 1.5 molar excess (0.37 mmol). The tube was sealed by a PTFE PCT MicroCap and secured in the metal MicroTube cassette. Then, the cassette with the reaction tubes was placed in the chamber of the Barocycler. The chamber was pressurized up to the desired pressure which was maintained for the desired time. After depressurizing the pressure chamber, the tubes were removed and the product was isolated by aqueous wash, neutralization with NaHCO_3_ and then extracted with ethyl acetate. The yield was determined by an Agilent 7250 GC-QTOF mass spectrometer operated in electron impact ionization (EI, 70 eV) mode using a 30m long DB-5 type column (J&W Scientific).

#### General procedure for the catalyst and solvent-free large-scale reaction of hexan-2,5-dione and aniline under high hydrostatic pressure

To a 100 mL low-density polyethylene (LDPE) bottle was added hexan-2,5-dione (65.14 g, 0.57 mol) and 1.0 equiv aniline (59.27 g, 0.57 mol) to fill up the entire reaction vessel, then the bottle was sealed by an LDPE screw cap. Afterward, the vessel was placed in the chamber compartment of a Pressure BioSciences HHP instrument with a 5 L chamber. The chamber was filled up with water and pressurized up to 3.8 kbar. The reaction mixture was reacted under 3.8 kbar at room temperature for 55 min. After removing the reaction vessel from the pressure chamber, the cap was removed and the solid material was collected and dried by a simple air-drying. After drying 96.92 g of the product was obtained. The purity of the product was determined by an Agilent 7250 GC-QTOF mass spectrometer operated in electron impact ionization (EI, 70 eV) mode using a 30m long DB-5 type column (J&W Scientific).

### Computational details

To determine the reaction volume (Δ*V*), the volumes of the starting materials and products were calculated separately using the Gaussian 09 program suite [48]. Geometry optimizations were performed using density functional theory (DFT) with the Becke three-parameter exchange and Lee–Yang–Parr correlation (B3LYP) functional [49], along with the 6–311++G(d,p) basis set for gas-phase calculations of all compounds. After completing the molecular geometry optimizations, vibrational frequency calculations were conducted to ensure that none of the optimized structures exhibited imaginary frequencies confirming that all structures corresponded to real local minima on the potential energy surface. For the volume calculations, the Gaussian keyword “volume” was used, which defines the molar volume as the region inside a contour of 0.001 electrons/Bohr^3^ density [50].

The reaction volume was calculated as:


[1] $$\Delta V=\;\Sigma V_{\text{products}}–\;\Sigma V_{\text{reactants}}$$

## Supporting Information

File 1 Materials and methods, spectral data of new compounds, and HRMS spectra of known products.

## Data Availability

All data that supports the findings of this study is available in the published article and/or the supporting information of this article.
