# Supplementary material for: High-pressure activation for the solvent- and catalyst-free syntheses of heterocycles, pharmaceuticals and esters
Source: Beilstein J Org Chem. 2025 Jul 2;21:1374–87. doi: 10.3762/bjoc.21.102 (PMC12230333; doi:10.3762/bjoc.21.102)
Supplement: File 1 — Materials and methods, spectral data of new compounds, and HRMS spectra of known products. [file Beilstein_J_Org_Chem-21-1374-s001.pdf]

BEILSTEIN JOURNAL OF ORGANIC CHEMISTRY

Supporting Information

for

High-pressure activation for the solvent- and catalyst-free syntheses of heterocycles, pharmaceuticals and esters

Kelsey Plasse, Valerie Wright, Guoshu Xie, R. Bernadett Vlocskó, Alexander Lazarev and Béla Török

Beilstein J. Org. Chem. **2025,** *21,* 1374–1387. doi:10.3762/bjoc.21.102

Materials and methods, spectral data of new compounds, and HRMS spectra of known products

License and Terms: This is a supporting information file under the terms of the Creative Commons Attribution License (https://creativecommons.org/licenses/by/4.0). Please note that the reuse, redistribution and reproduction in particular requires that the author(s) and source are credited and that individual graphics may be subject to special legal provisions.
The license is subject to the *Beilstein Journal of Organic Chemistry* terms and conditions: (https://www.beilstein-journals.org/bjoc/terms)

Table of contents

1. Materials and methods S2

2. Spectral data of new compounds S3

3. HRMS spectra of known products S6

4. References S13

1. Materials and methods

Materials: All substrates (aldehydes, hydrazones, alcohols, *p*-aminophenol, salicylic acid, acetic anhydride, acetic acid, hexan-2,5-dione, aniline, hexylamine and 2-phenylethylamine) were purchased from Aldrich and used without any purification. $CDCl_3$ and DMSO-d_6 used as a solvent (99.8%) for NMR studies was an Aldrich product. Ethyl acetate used to dissolve the product for GC–MS analysis (minimum purity of 99.5%) was purchased from ThermoFisher Scientific. The small scale reactor tubes were made of teflon and obtained from Pressure BioSciences Inc. while the larger scale bulbs and bottles used as reaction vessels were made of low-density polyethylene and purchased from ThermoFisher Scientific.

Analysis: The ^{1}H and ^{13}C spectra of new compounds were obtained on a 400 MHz Agilent MM2 NMR spectrometer, in $CDCl_3$ with either using the signal of tetramethylsilane or the residual solvent signal as reference. The temperature was 25 °C (accuracy ±1 °C). All products were known compounds and the NMR spectra were in agreement with earlier sources. The mass spectrometric identification and purity and yield determination (referred to as GC yields) of the products have been carried out by using an Agilent 7250 GC-QTOF mass spectrometer operated in electron impact ionization (EI, 70 eV) mode using a 30 m long DB-5 type column (J&W Scientific).

2. Spectra of the new products:

The compounds were known products, except for 1,3-dihydrobenzimidazoles **3b** and **3d**. The characterization for these compounds is provided below, while the HRMS spectra are provided for the other known products.

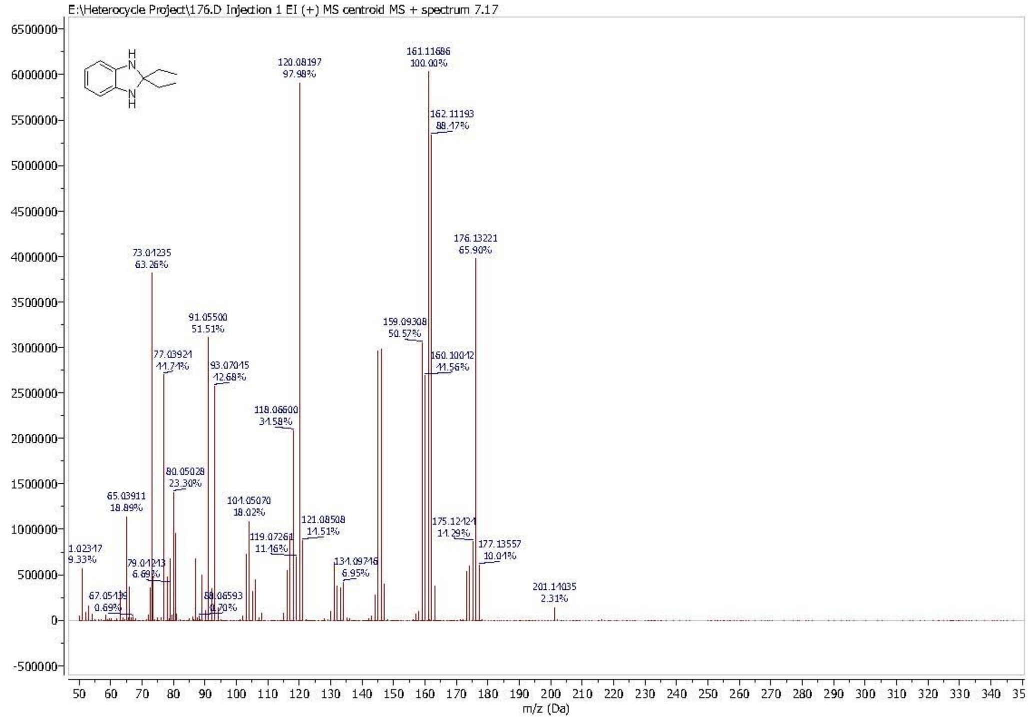

Figure S1. HRMS spectrum of 2,2-diethyl-2,3-dihydro-1*H*-benzo[*d*]imidazole (**3b**). (EI): m/z: calcd. for $C_{11}H_{16}N_2$: 176.1313, found: 176.1322

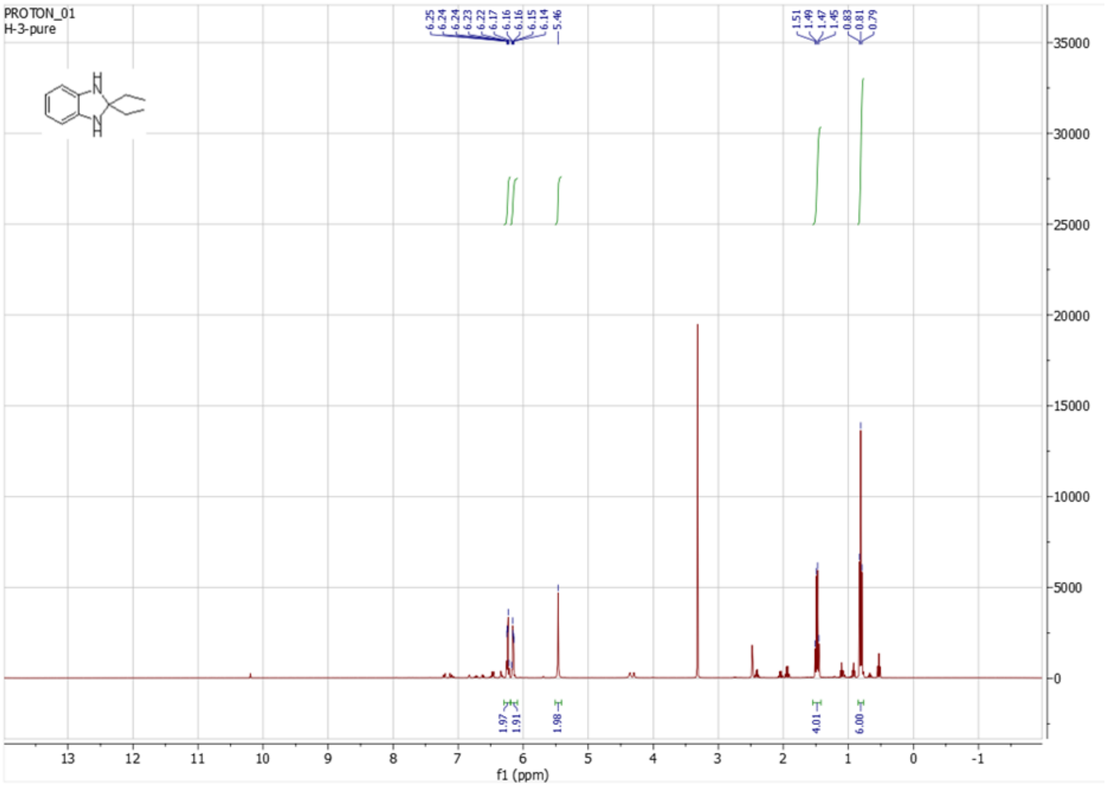

Figure S2. ^{1}H NMR spectrum of 2,2-diethyl-2,3-dihydro-1*H*-benzo[*d*]imidazole (**3b**).

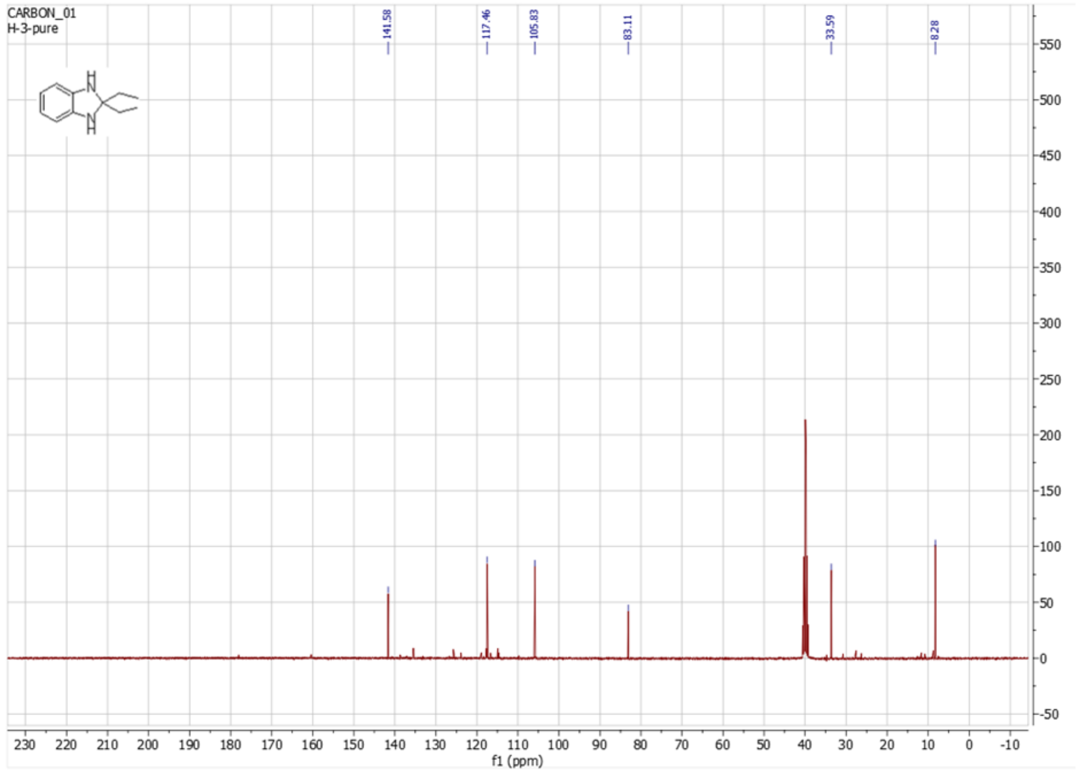

Figure S3. ^{13}C NMR spectrum of 2,2-diethyl-2,3-dihydro-1*H*-benzo[*d*]imidazole (**3b**).

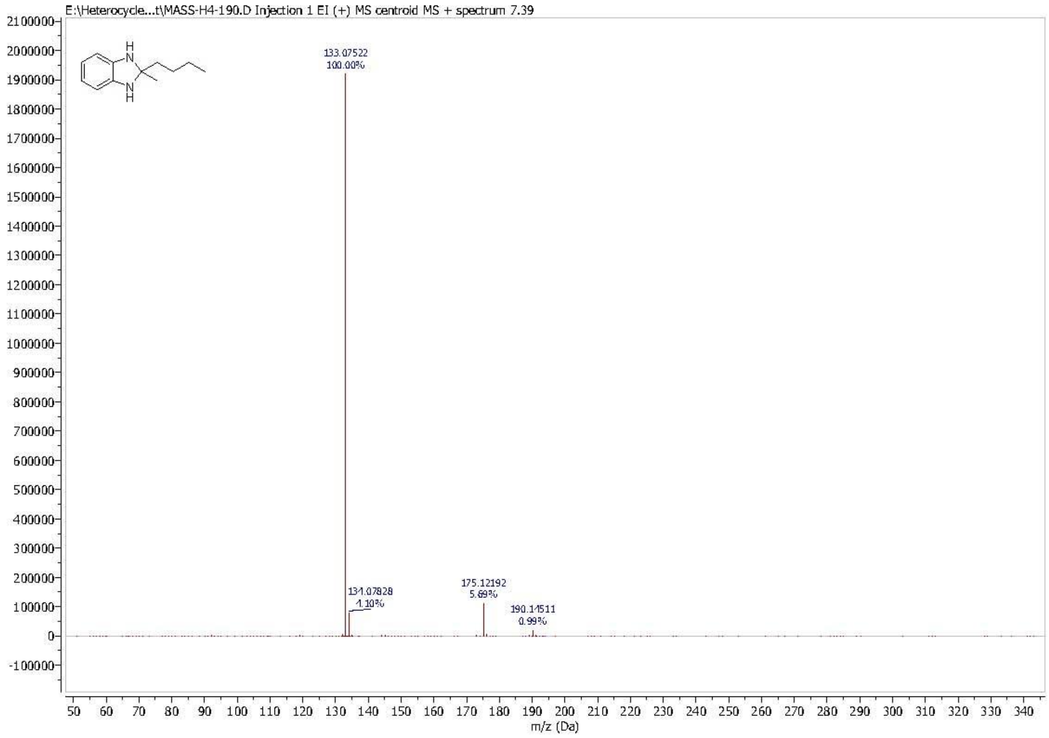

Figure S4. HRMS spectrum of 2-butyl-2-methyl-2,3-dihydro-1*H*-benzo[*d*]imidazole (**3d**). (EI): m/z: calcd. for $C_{12}H_{18}N_2$: 190.1470, found: 190.1451

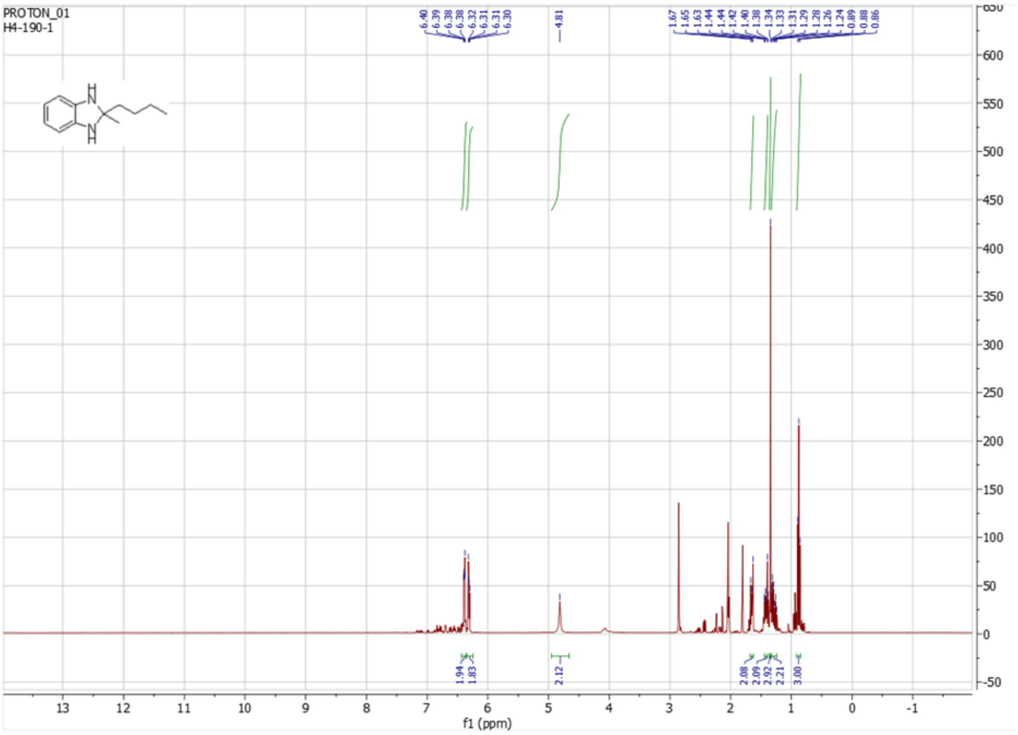

Figure S5. ^{1}H NMR spectrum of 2-butyl-2-methyl-2,3-dihydro-1*H*-benzo[*d*]imidazole (**3d**).

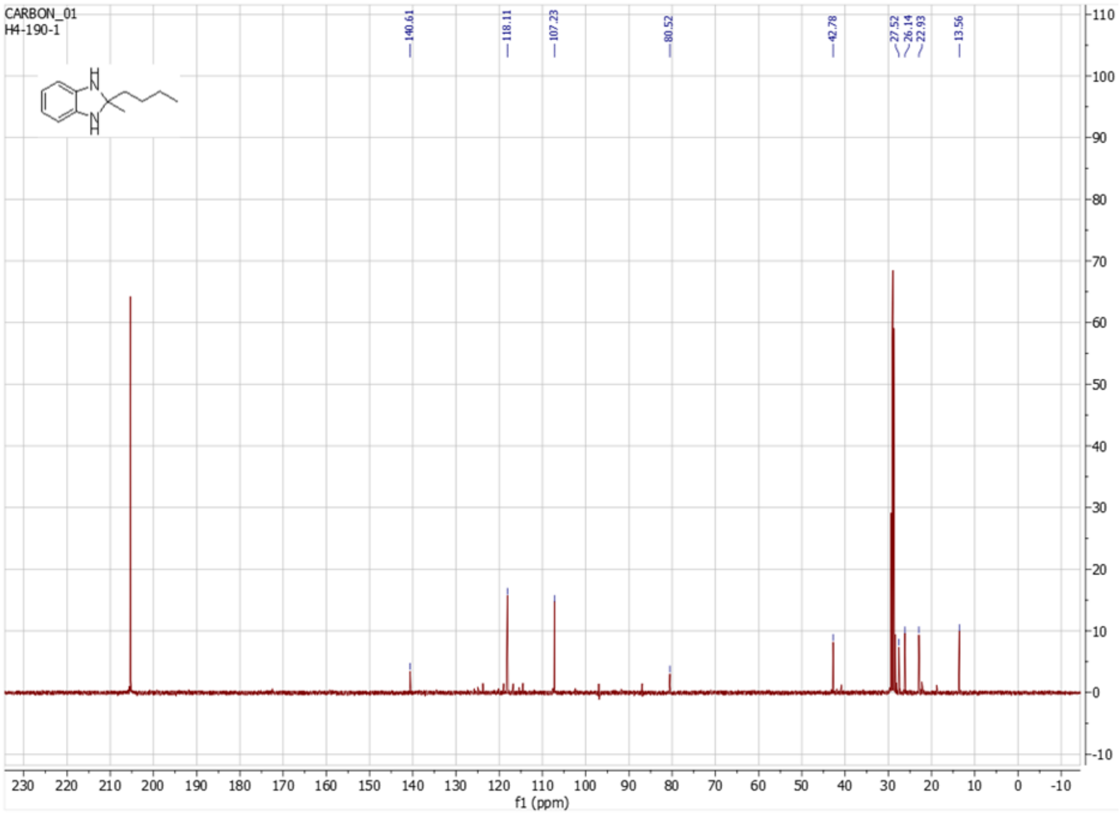

Figure S6. ^{13}C NMR spectrum of 2-butyl-2-methyl-2,3-dihydro-1*H*-benzo[*d*]imidazole (**3d**).

3. HRMS spectra of known products

The data are in agreement with the relevant literature and is within the 5ppm expected accuracy to the calculated values.

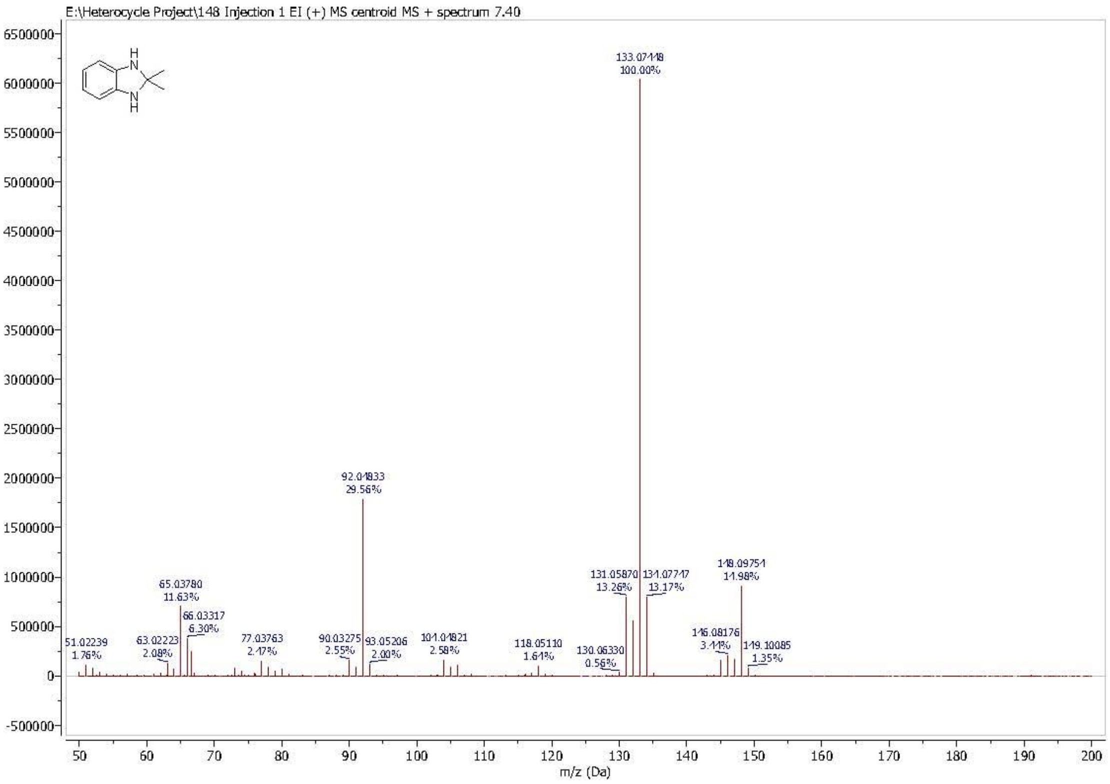

Figure S7. HRMS spectrum of 2,2-dimethyl-2,3-dihydro-1*H*-benzo[*d*]imidazole (**3a**).[1]

(EI): m/z: calcd. for $C_9H_{12}N_2$: 148.1000, found: 148.0975

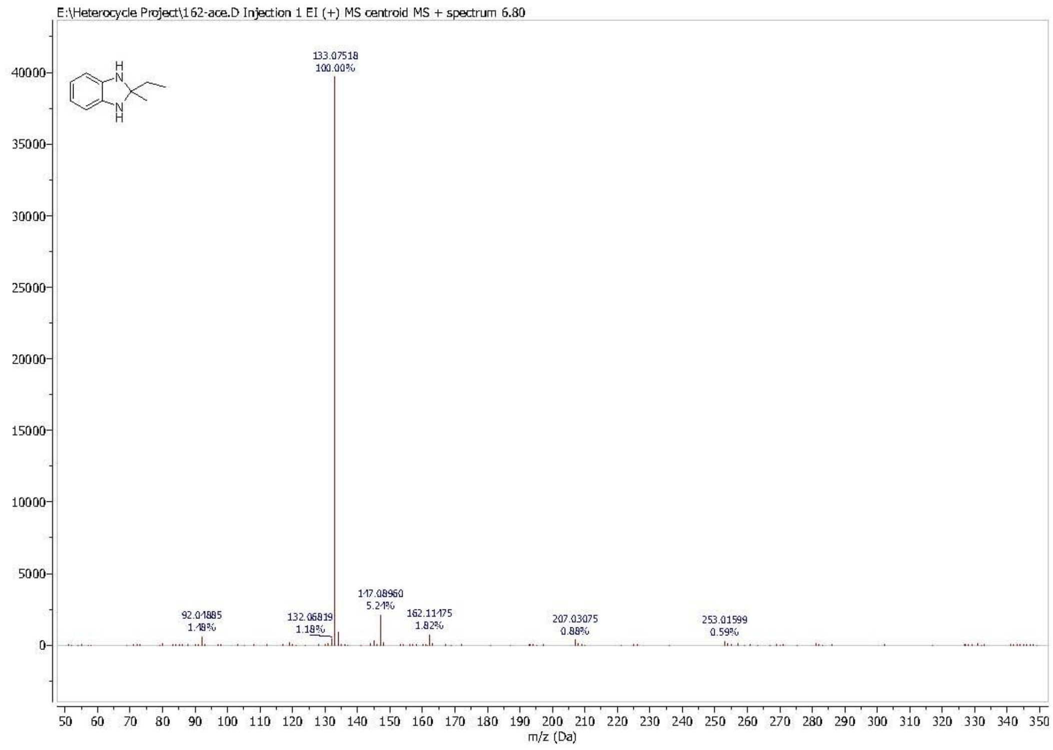

Figure S8. HRMS spectrum of 2-ethyl-2-methyl-2,3-dihydro-1*H*-benzo[*d*]imidazole(**3c**).[2]

(EI): m/z: calcd. for $C_{10}H_{14}N_2$: 162.1157, found: 162.1147

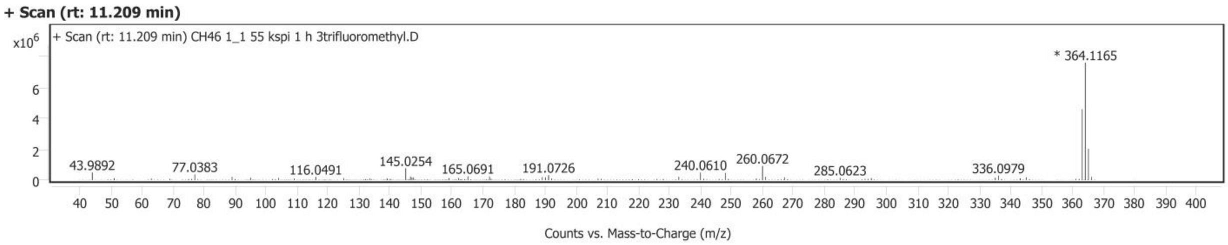

Figure S9. HRMS spectrum of 3,5-diphenyl-1-(3-(trifluoromethyl)phenyl)-1*H*-pyrazole (**6a**).[3]

(EI): m/z: calcd. for $C_{22}H_{15}F_3N_2$: 364.1187, found: 364.1165

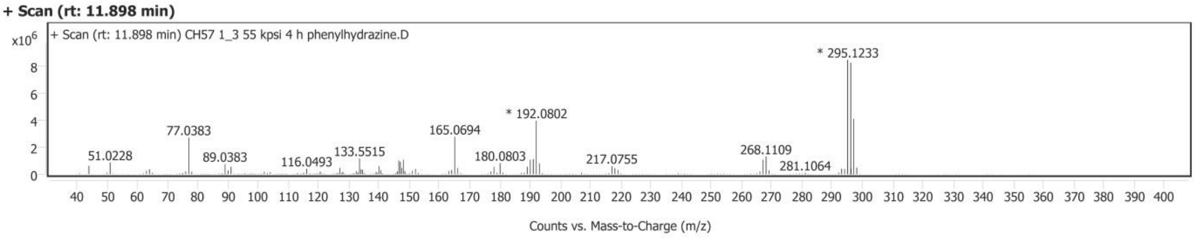

Figure S10. HRMS spectrum of 1,3,5-triphenyl-1*H*-pyrazole (**6b**).[3]

(EI): m/z: calcd. for $C_{21}H_{15}N_2$: 295.1230, found: 295.1233

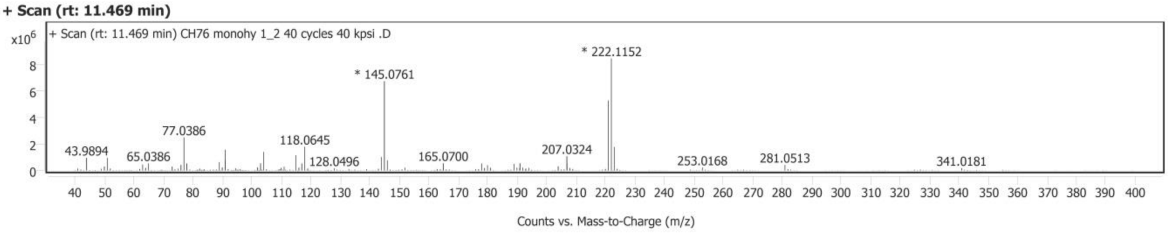

Figure S11. HRMS spectrum of 3,5-diphenyl-4,5-dihydro-1*H*-pyrazole (**6c**).[4]

(EI): m/z: calcd. for $C_{15}H_{14}N_2$: 222.1157, Found: 222.1152

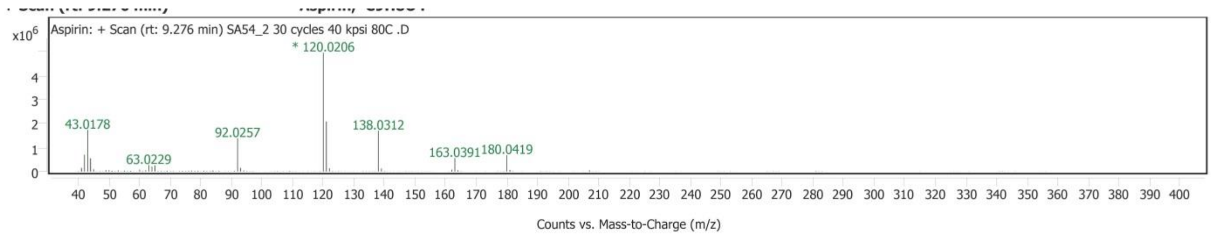

Figure S12. HRMS spectrum of acetylsalicylic acid (**9**).[5]

(EI): m/z: calcd. for $C_9H_8O_4$: 180.0423, found: 180.0419

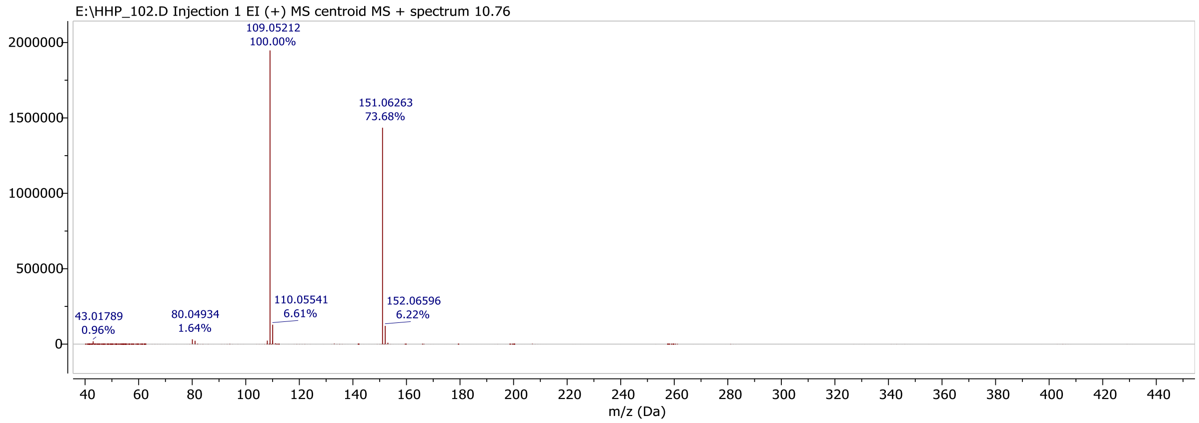

Figure S13. HRMS spectrum of acetaminophen (**11**).[6]

(EI): m/z: calcd. for $C_8H_9NO_2$: 151.0633, found: 151.0626

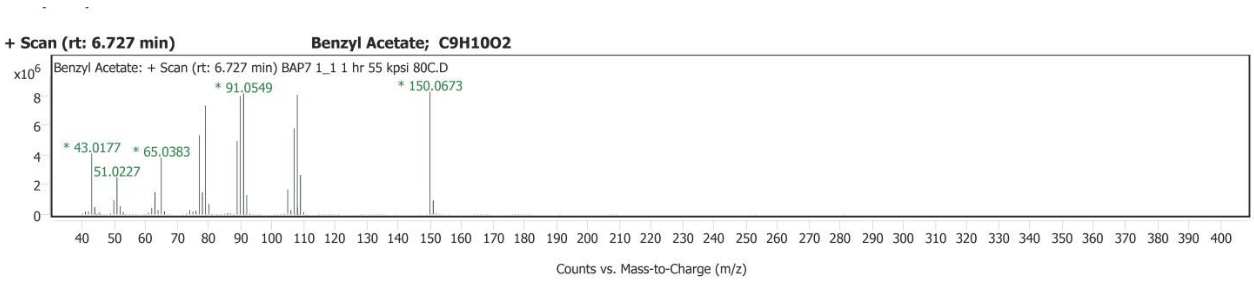

Figure S14. HRMS spectrum of benzyl acetate (**14a**).[7]

(EI): m/z: calcd. for $C_9H_{10}O_2$: 150.0681, found: 150.0673

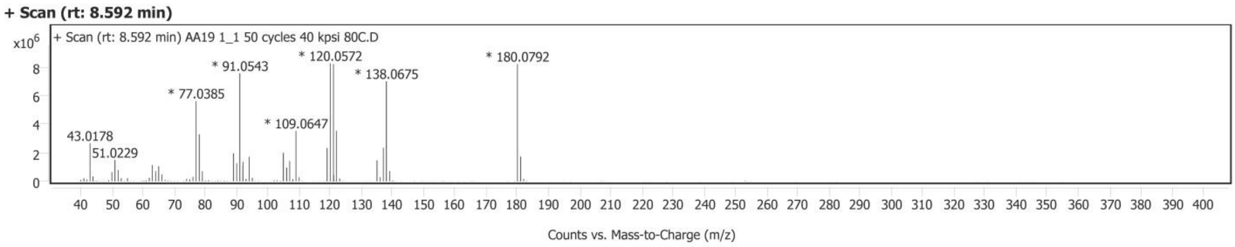

Figure S15. HRMS spectrum of 4-methoxybenzyl acetate (anisyl acetate, **14b**).[8]

(EI): m/z: calcd. for $C_{10}H_{12}O_3$: 180.0786, found: 180.0792

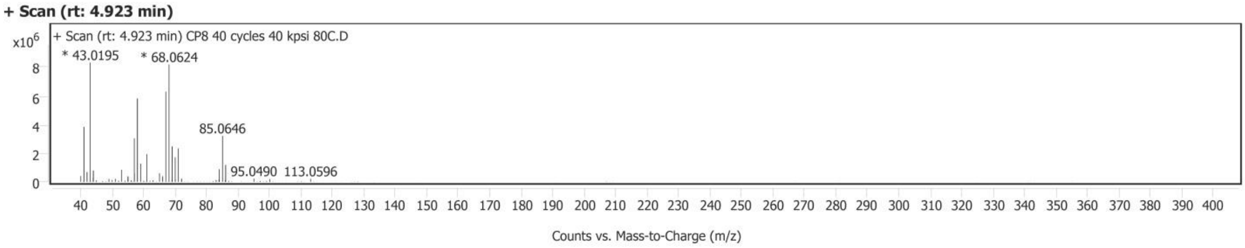

Figure S16. HRMS spectrum of cyclopentyl acetate (**14c**).[9]

(EI): m/z: calcd. for $C_6H_9O_2$: 113.0597, Found: 113.0596

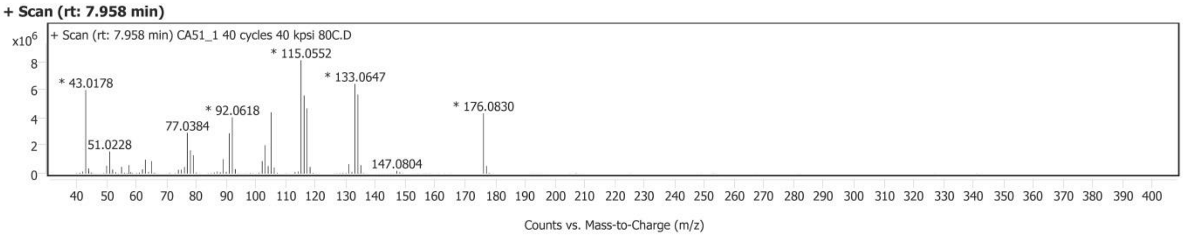

Figure S17. HRMS spectrum of cinnamyl acetate (**14d**).[10]

(EI): m/z: calcd. for $C_{11}H_{12}O_2$: 176.0837, found: 176.0830

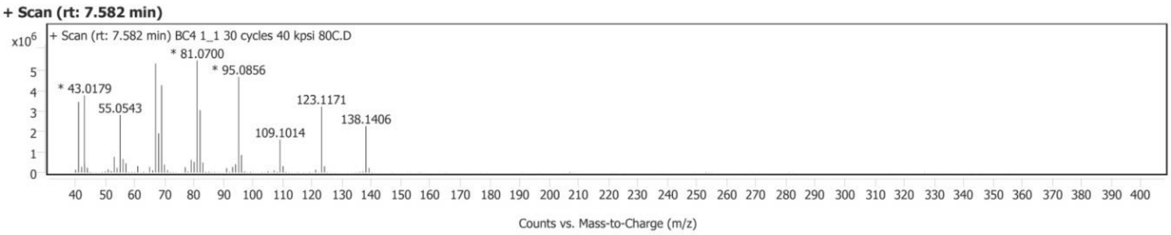

Figure S18. HRMS spectrum of β-citronellyl acetate (**14e**).[11]

(EI): m/z: calcd. for $C_{10}H_{18}^+$, Calc.: 138.1409, Found: 138.1406

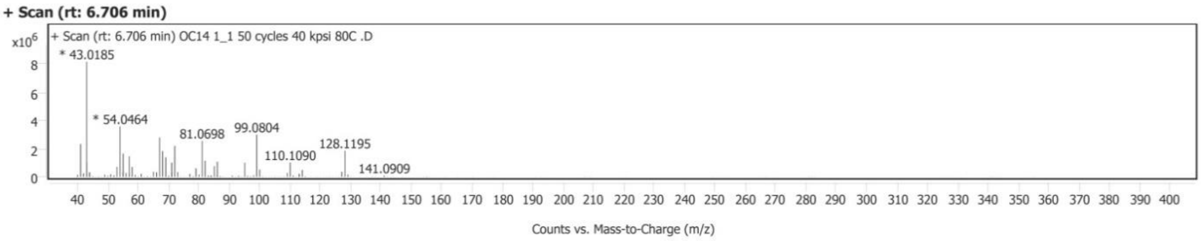

Figure S19. HRMS spectrum of oct-1-en-3-ol acetate (**14f**).[12]

(EI): m/z: calcd. for $C_5H_7O_2$: 99.0446, found: 99.0804

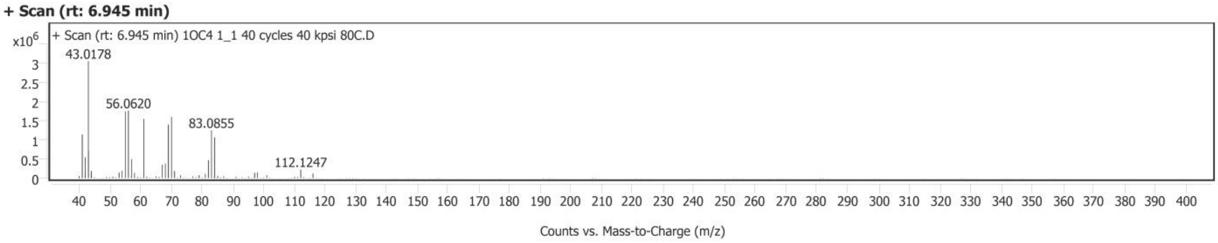

Figure S20. HRMS spectrum of octyl acetate (**14g**)[12]

(EI): m/z: calcd. for C_8H_{16} : 112.1252, Found: 112.1247

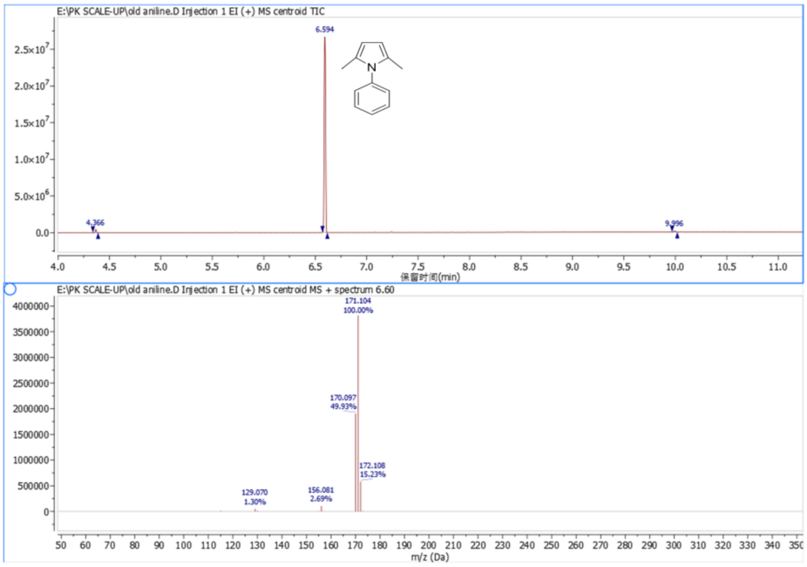

Figure S21. HRMS spectrum of 1-phenyl-2,4-dimethylpyrrole (**17a**)[13]

(EI): m/z: calcd. for $C_{12}H_{13}N$: 171.104, found: 171.104

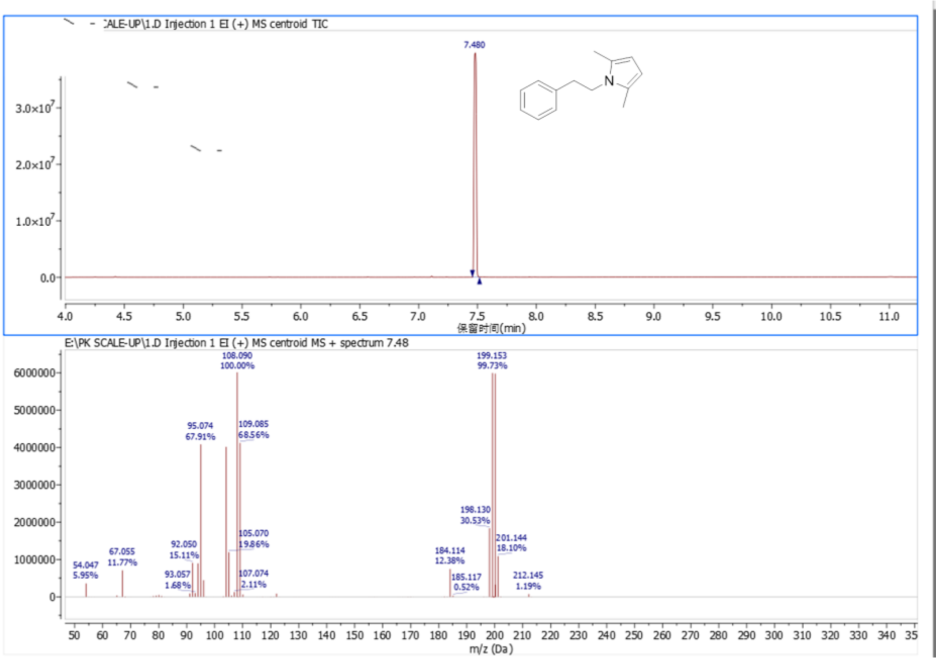

Figure S22. HRMS spectrum of 2,5-dimethyl-1-phenethyl-1*H*-pyrrole (**17b**)[15]

(EI): m/z: calcd. for $C_{14}H_{17}N$: 199.136, found: 199.153

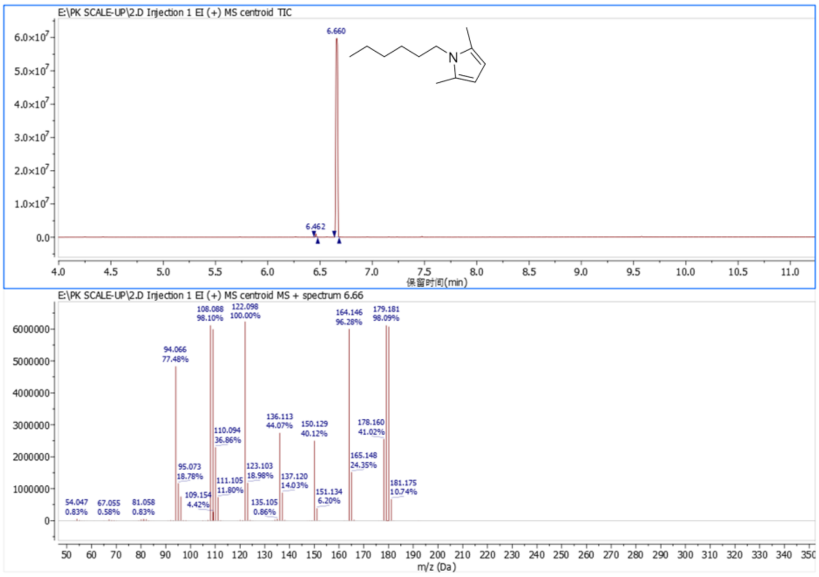

Figure S23. HRMS spectrum of 1-hexyl-2,4-dimethylpyrrole (**17c**)[15]

(EI): m/z: calcd. for $C_{12}H_{21}N$: 179.167, found: 179.181

4. References

[1] M. Godino-Ojer, L. Milla-Diez, I. Matos, C. J. Duran-Valle, M. Bernardo, I. M. Fonseca, E. Perez Mayoral, *ChemCatChem* **2018**, *10*, 5215-5223.

[2] S. Nemeth, L. Simandi, *J. Mol. Catal*. **1982**, *14*, 87-93.

[3] S. M. Landge, A. Schmidt, V. Outerbridge, B. Török, *Synlett* **2007**, 1600-1604.

[4] Ju, Yuhong; Varma, Rajender S. *J. Org. Chem*. **2006** , *71*, 135–141.

[5] Allen F., Pon A., Greiner R., Wishart D. *Anal Chem*. **2016,** *88*, 7689-7697.

[6] W.A. Garland, K.C. Hsiao, E.J. Pantuck, A.H. Conney, *J. Pharm. Sci*. **1977**, *66*, 340-344.

[7] D. CameronR. G. Cooks, *J. Am. Chem. Soc*. **1979**, *101*, 3162-3168.

[8] Kishor P. Dhake, Pawan J. Tambade, Ziyauddin S. Qureshi, Rekha S. Singhal, and Bhalchandra M. Bhanage, *ACS Catalysis* **2011**, *1*, 316-322.

[9] Rao, C.B., Rao, D.C., Babu, D.C. and Venkateswarlu, Y. *Eur. J. Org. Chem*. **2010**, 2855-2859.

[10] Débieux, J.-L., Cosandey, A., Helgen, C. and Bochet, C.G. *Eur. J. Org. Chem*., **2007**, 2073-2077.

[11] S.-M. Seo, J. Kim, S.-G. Lee, C.-H. Shin, S.-C. Shin, I.-K. Park, *J. Agric. Food Chem*. **2009**, *57*, 15, 6596–6602.

[12] M. Barbero, S. Cadamuro, S. Dughera, P. Venturello, *Synthesis* **2008**, 3625-3632.

[13] G. Xie, A. Lazarev, B. Török, *Green Chem*. **2023**, *25*, 1582–1587
